# Area-specific covid-19 effects on health services utilization in the Democratic Republic of the Congo using routine health information system data

**DOI:** 10.1186/s12913-023-09547-9

**Published:** 2023-06-03

**Authors:** Gustavo Angeles, Hannah Silverstein, Matt Worges, David R. Hotchkiss, Janna M. Wisniewski, Paul Samson Lusamba Dikassa, William Weiss, Karar Zunaid Ahsan

**Affiliations:** 1 Department of Maternal and Child Health, Gillings School of Global Public Health, University of North Carolina at Chapel Hill, Chapel Hill, NC USA; 2 Department of International Health and Sustainable Development, School of Public Health and Tropical Medicine, Tulane University, New Orleans, LA USA; 3 Tulane International LLC, Kinshasa, Democratic Republic of the Congo; 4 Kinshasa School of Public Health, The University of Kinshasa, DRC Kinshasa, Democratic Republic of the Congo; 5 Department of International Health, The John Hopkins University, Baltimore, MD USA; 6 Public Health Leadership Program, Gillings School of Global Public Health, University of North Carolina at Chapel Hill, NC Chapel Hill, USA

**Keywords:** COVID-19, Health facility data, Health services utilization, Universal health coverage, Health management information systems (HMIS), DHIS2, Maternal and child health, Democratic Republic of the Congo (DRC)

## Abstract

**Background:**

Since March 2020, the COVID-19 pandemic has shocked health systems worldwide. This analysis investigated the effects of the pandemic on basic health services utilization in the Democratic Republic of the Congo (DRC) and examined the variability of COVID effects in the capital city Kinshasa, in other urban areas, and in rural areas.

**Methods:**

We estimated time trends models using national health information system data to replicate pre-COVID-19 (i.e., January 2017–February 2020) trajectories of health service utilization, and then used those models to estimate what the levels would have been in the absence of COVID-19 during the pandemic period, starting in March 2020 through March 2021. We classified the difference between the observed and predicted levels as the effect of COVID-19 on health services. We estimated 95% confidence intervals and *p*-values to examine if the effect of the pandemic, nationally and within specific geographies, was statistically significant.

**Results:**

Our results indicate that COVID-19 negatively impacted health services and subsequent recovery varied by service type and by geographical area. COVID-19 had a lasting impact on overall service utilization as well as on malaria and pneumonia-related visits among young children in the DRC. We also found that the effects of COVID-19 were even more immediate and stronger in the capital city of Kinshasa compared with the national effect. Both nationally and in Kinshasa, most affected services had slow and incomplete recovery to expected levels. Therefore, our analysis indicates that COVID-19 continued to affect health services in the DRC throughout the first year of the pandemic.

**Conclusions:**

The methodology used in this article allows for examining the variability in magnitude, timing, and duration of the COVID effects within geographical areas of the DRC and nationally. This analytical procedure based on national health information system data could be applied to surveil health service disruptions and better inform rapid responses from health service managers and policymakers.

**Supplementary Information:**

The online version contains supplementary material available at 10.1186/s12913-023-09547-9.

## Background

With cases reported in more than 110 countries and territories [1], the World Health Organization declared the COVID-19 outbreak a pandemic on March 11, 2020 [2, 3]. After the formal declaration, countries took various steps to prevent the outbreak of COVID-19 by mandating social distancing, closing of schools and businesses, and implementing national or regional lockdowns. Since March 2020, the COVID-19 pandemic has affected societies in countless ways, like travel restrictions, health personnel shortages, and global supply chain interruptions [4–7]. Such disruptions also affected the availability, access, and utilization of essential health services in both developed and developing countries.

A review in 2021 found that most research on the effects of COVID-19 on health services not related to COVID-19 focused on high-income countries [4]. There exist few studies in low- and middle-income countries (LMIC) indicating that COVID-19 had negative effects on the overall health services use during the early months of the pandemic [5–9]. These studies, however, have either used non-robust, descriptive analyses [5, 10–12] or considered pandemic period data from a relatively short time frame, only examining COVID-19 effects through mid-2020 [5, 7–11]. Since the pandemic has been ongoing for nearly two years, the effects on health services use may have evolved over time, which would be important to examine for informing the also evolving policy response. Another study investigated COVID-effects on health service utilization over a longer period of time in 18 LMICs. Results show that many countries experienced reductions in outpatient department volume, but the types of maternal and child health services most effected depended on the context [13]. Since COVID-19 effects on health systems varied between countries, there were likely service disruptions within countries as well. Large cities were most immediately affected by COVID-19 in terms of closures and cases, so it is also important to examine how the pandemic may have had different effects across geographic areas within a country.

This study estimates both national- and subnational-level effects of COVID-19 in the Democratic Republic of the Congo (DRC) on key basic health services, including maternal and child health (MCH), family planning (FP), and immunizations, using data collected by routine health information system before and during the first full year of COVID-19. Because there were other outbreaks during early 2020, including Ebola virus and measles, combined with a strained healthcare system and large populations in urban areas, the DRC was considered highly vulnerable to COVID-19 at the start of the pandemic [7]. The first case of COVID-19 in the DRC was announced on March 10, 2020, and the country President declared a health emergency on March 19. The first lockdown measures were in Kinshasa. The strictest restrictions were in the Gombe neighborhood, considered the epicenter of the epidemic at the time. Restrictions in Gombe lasted about four months [7, 14]. Across the DRC, province-level committees established local responses to COVID-19, such as enforcing gathering restrictions that attract large numbers of people, which would include vaccination and other health related sessions. Many cities also had checkpoints to take temperatures of individuals upon entrance, and in some circumstances, travel was restricted in and out of provinces as well [14].

This background indicates that there was great potential for COVID-19 to impact the health system within the DRC. Therefore, this research had two main objectives: to examine how the COVID-19 pandemic has affected 1) reporting of health service utilization and 2) utilization of health services. Additionally, the diverse responses to the COVID-19 outbreak across the DRC likely resulted in differential effects on the health systems in different areas. In this analysis, we investigate usage patterns before and during the COVID-19 pandemic for selected health services at the national-level and for the following subnational areas: Kinshasa, the capital city; other urban areas; and rural areas. We developed models using data from the pre-pandemic period replicating the trajectories over time of total and average health service utilization, as well as the number of facilities reporting such services. From these models, we estimate what health service utilization levels and patterns would have been in the absence of COVID-19 from March 2020 through March 2021, the first full year of the pandemic. We define the “COVID-19” effect as the gap between the observed utilization during the pandemic and the level we predict would have happened without COVID-19.

## Methods

### Data

This study is a secondary analysis using data available through the routine health information system (RHIS) in the DRC, known Système National d’Information Sanitaire, which is the national District Health Information Software version 2 (DHIS2) platform for health information management [12]. DHIS2 was introduced in the DRC for RHIS in 2014, and it expanded throughout the country during by 2016/2017 [15–17]. To ensure more consistent coverage and quality, we use facility-level DHIS2 data from January 2017 through March 2021 on ten selected essential health services (see Table 1). Because the DHIS2 datasets were aggregated by the system at the facility level, data were anonymized and contained no personal identifiers prior to analysis. Datasets were also cleaned prior to all analysis using a specific process to identify and remove outliers: first, we generated facility-level time series graphs to identify extreme values for each service type, and then estimated the within-unit mean and standard deviation for each service. These graphs informed rules for removing potential outliers, for example, eliminating values exceeding 6 standard deviations above the unit-level mean. During the cleaning process, we found less than 0.2% of data points for each service to be the outliers.

**Table 1:** Summary of dataset and variables

**Country**	**RHIS system used**	**Data unit-level**	**Time range**	**Health Services**
DRC	District Health Information Software 2 (DHIS2)	Facility (*N*=16,668 in March 2021)	January 2017-March 2021	All cases received (total facility visits) 1^st^ antenatal visits (ANC1) 4^th^ antenatal visits (ANC4) Facility deliveries Family planning visits Diarrhea cases (age<5 years) Malaria cases (age<5 years) Pneumonia cases (age<5 years) Measles vaccine doses 1^st^ pentavalent vaccine doses

### Measures

A variety of basic, essential health services was used in this analysis. These services fall into three categories: maternal and infant health (first and fourth antenatal care [ANC] visits, facility deliveries, and child immunization); outpatient visits for young child illness (under-5 cases of diarrhea, malaria, and pneumonia); and family planning visits. Additionally, we included one indicator that captures the overall service utilization, which we refer to as all cases received.

There were four ways each service was analyzed, which we refer to in this paper as outcome types. The four outcomes types for each service are the following: 1) the number of facilities reporting; 2) the total number of service visits reported; 3) the adjusted visit total, accounting for COVID-19’s effects on the number of facilities reporting; and 4) the average number of visits per facility.

To answer the first research question, whether COVID-19 affected reporting, we generated variables of the first type listed, counting the number of facilities reporting any number of visits (prior to the data cleaning) for each service each month. For answer the second question, whether COVID-19 affected health service utilization, we generated monthly estimates for the latter three outcome types listed above.

### Analytical approach

There were two processes involved in analyzing the data, which have been described previously and applied in similar work [18–23]. The first process, which we called the ‘internal process’, examined pre-COVID patterns and fitted a time trends model that most closely replicated those observed pre-COVID trajectories of service utilization over time. In the second process, we estimated the levels of service use that would have been observed had COVID-19 not occurred during the COVID-19 months. To do this, we extended the models to fit pre-COVID patterns, derived from the first process, through the COVID-19 period. The difference between the observed level and the predicted for each time point is classified as the “COVID-19 effect”.

The first COVID-19 case in the DRC was reported on 10 March 2020, and the first death occurred a few days later [3]. All international flights from the DRC were suspected from 19 March 2020, and the government declared a state of emergency on 24 March 2020, closing borders, schools, restaurant, bars, and places of worship [7]. Because the DRC first reported COVID-19 cases were in early March 2020 and experienced subsequent lockdowns later in the same month, the pre-COVID period was defined as occurring between January 2017 through February 2020. The COVID-19 period was defined as occurring between March 2020 through March 2021. Note that the DHIS2 in the DRC was introduced in 2016, but we excluded data from this first year based on recommendations from local partners, to ensure more consistent data quality and availability throughout time series. Starting the pre-COVID period in 2017 also allowed models to better capture seasonal patterns in health service utilization and reporting since the dataset included three complete years of data before the pandemic.

#### Descriptive analysis and examining pre-COVID patterns

We undertook an initial descriptive analysis to answer the first question: did COVID-19 affect reporting? We counted the number of facilities reporting each month for each service from January 2017 until March 2021. We examined the patterns of facility reporting over time and if there were disruptions in facilities reporting services from March 2020 onward, after the onset of the COVID-19 pandemic.

We carried out model estimation procedures to answer the second research question: did COVID-19 affect service utilization? For any given service, we compared several pieces of information to make final determinations on which model to use for estimating the COVID effect. Before estimating any models, we undertook several steps to determine the functional form of time on service utilization before COVID-19, detailed in supplementary material S1. For services where reporting rates fluctuated, particularly during COVID-19 months, we also applied the same model fitting procedure to the number of facilities reporting as an outcome. We determined that a linear trend for time best fit the pattern for almost all services in the DRC.

#### Replicating pre-COVID patterns

We then compared the estimates from various regression models to determine which models best fit the reported levels and trajectories of each indicator and outcome type. The functional form analysis indicated that most services and outcomes followed a linear time trend, however, to confirm these conclusions we also compared these results with quadratic models as well. For both quadratic and linear functional forms, we explored a variety of regression approaches, including ordinary least squares, fixed effects, random effects, and autoregressive models. We included seasonal and yearly controls in addition to the continuous time-series variable in each model. We also explored quarterly and monthly dummy variables as seasonal controls. Additionally, for each service, the total utilization model controlled for the number of facilities reporting. More details on how model fit was assessed can be found in supplementary material S2.

Linear OLS models with monthly controls best replicated pre-COVID-19 trajectories for all total service utilization for the number of facilities reporting in the DRC. In the models, $${time}_{t}$$ is the number of months since January 2017 up to t, $${Month}_{t}$$ is an array of monthly dummy variables where January is the reference month, $${year}_{t}$$ is an array of yearly variables controlling for 2018 and 2019, $${NFacilities}_{t}$$ is the number of facilities reporting at time *t*, and $${\varepsilon }_{t}$$ is the error terms. Equation 1 presents the model replicating total service utilization of service *K* at time *t* ($${TotalUseK}_{t})$$ during the pre-COVID period (January 2017–February 2020; *t*<39):

1 $${TotalUseK}_{t<39}={\alpha }_{0}+{\alpha }_{1}{time}_{t}+{\varvec{\beta}}{\left(Month\right)}_{t}+{\varvec{\gamma}}{\left(year\right)}_{t}+\delta {NFacilitiesK}_{t}+{\varepsilon }_{t}$$

As mentioned, the DRC data was at the facility level and there was more reporting variability. Therefore, we also modelled the number of facilities reporting (${NFacilitiesK}_{t<39}$) for service *K* at time *t* during the pre-COVID time period using specification presented in Eq. 2.

2 $${NUnitsK}_{t<39}={\alpha }_{0}+{\alpha }_{1}{time}_{t}+{\varvec{\beta}}{\left(Month\right)}_{t}+{\varvec{\gamma}}{\left(Year\right)}_{t}+{\varepsilon }_{t}$$

We found linear OLS with cluster-adjusted standard errors best replicated average service use in the DRC. In Eq. 3, we define ${UseK}_{jt}$ as the number of visits for service *K* at facility *j* at time *t,* and ${\varepsilon }_{jt}$ is the error term to estimate national averages of service provision per facility for each service:

3 $${UseK}_{jt}={\alpha }_{0}+{\alpha }_{1}{time}_{t}+{\varvec{\beta}}{\left(Month\right)}_{t}+{\varvec{\gamma}}{\left(Year\right)}_{t} + { \varepsilon }_{jt}$$

Additionally, we estimated area-specific models to see how trajectories varied across urban areas in Kinshasa, other urban areas, and rural areas using interaction models, stratified by area category, to estimate area-specific trajectories. Equation 4 represents how area-specific predictions were obtained, where K represents a service utilization at time *t* for facility* j*:

4 $$\begin{array}{c}{K}_{jt}={\alpha }_{0}+{\alpha }_{1}{time}_{t}+{\varvec{\beta}}{\left(Month\right)}_{t}+{\varvec{\gamma}}{\left(Year\right)}_{t}\\ +{OtherUrban}_{j}*\left[{\delta }_{0}+{\delta }_{1}{time}_{t}+\theta {\left(Month\right)}_{t}+\boldsymbol{\vartheta }{\left(Year\right)}_{t}\right]\\ +{Rural}_{j}*\left[{\tau }_{0}+{\tau }_{1}{time}_{t}+\varphi {\left(Month\right)}_{t}+{\varvec{\omega}}{\left(Year\right)}_{t}\right]+{\varepsilon }_{jt}\end{array}$$

Where ${OtherUrban}_{j}$ is a dummy variable indicating that facility* j* is located in other urban areas, and ${Rural}_{j}$ is a dummy indicating whether the facility is in rural areas. Note that urban Kinshasa is the reference location category in model (4). Similar models were estimated for the number of facilities reporting and the total.

#### Quantifying the effects of COVID-19

Upon deciding on model specifications, we then predicted levels of service utilization and standard errors for the entire time series, including the COVID-19 period. Predicted values from the models during the COVID-19 months represent the levels of service utilization that would have been observed had COVID-19 not occurred, maintaining the pre-COVID-19 time trends and seasonality patterns. The estimated COVID-19 effect is the difference between the reported and the predicted levels. Using the predicted standard errors, we estimated 95% confidence intervals and *p*-values to test the null hypothesis that the COVID-19 effect was not significantly different than zero for a given month in the COVID-19 period. More details on how we tested hypotheses and calculated significance can be found in Supplementary Material S3.

**Fig. 1:**
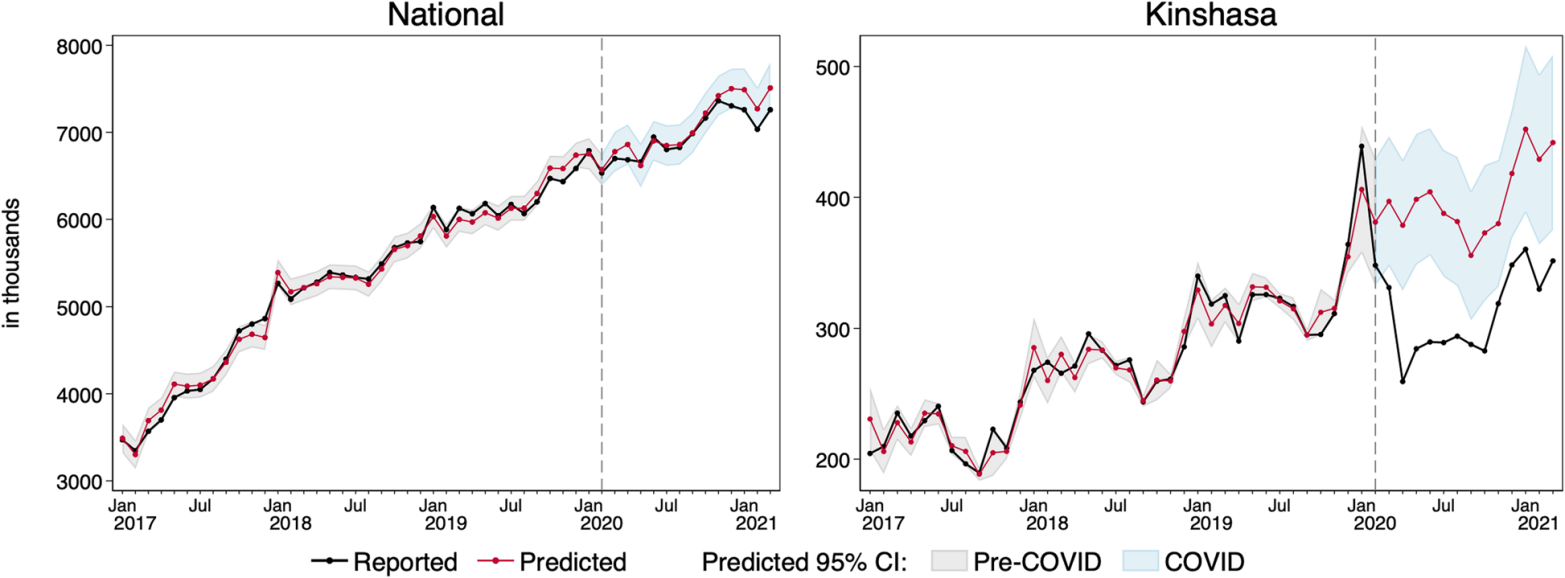
Observed and predicted total for all cases received in the DRC, nationally and in Kinshasa

Figure 1 presents an example of one such model, which displays observed and predicted levels from the total model for all cases received in the DRC, both nationally and in Kinshasa. The black line is the observed total cases. The red line is the respective values predicted by the time trends model. The vertical gray dashed line at February 2020 divides the time series into the pre-COVID period (January 2017–February 2000) and the COVID-19 period (March 2020–March 2021). The gray shaded area represents the 95% confidence interval for that model in the pre-COVID period. There are relatively few time points in the pre-COVID period that exceed this gray shaded area reflecting that this model fits the observed trajectories well, both nationally and in Kinshasa.

The predicted 95% confidence interval was used to determine the COVID-19 effect. In Fig. 1, the blue area represents the 95% confidence interval during COVID-19 months, had COVID-19 not occurred. Time points where the black line extends beyond the shaded blue area are months where the reported levels do not fall within the predicted range. Using Fig. 1 as an example, COVID-19 significantly impacted the total cases seen in Kinshasa for the entire pandemic period.

Additionally, there were some fluctuations in the number of facilities reporting in the COVID-19 period. This provided evidence that COVID-19 may have affected reporting as well as service utilization. Therefore, we estimated adjusted COVID effects for the total service utilization models which used the predicted number of facilities reporting in the COVID-19 period ($${\widehat{NUnits}}_{t})$$ instead of the observed number of facilities reporting ($${NUnits}_{t})$$, represented by the following equation:

5 $${\widehat{TotalUseK}}_{t>38}={\widehat{\alpha }}_{0}+{\widehat{\alpha }}_{1}{time}_{t}+\widehat{{\varvec{\beta}}}{\left(Month\right)}_{t}+\widehat{{\varvec{\gamma}}}{\left(year\right)}_{t}+\widehat{\delta }{\widehat{NUnits}}_{t>38}$$

The adjusted models provide the complete COVID-19 effect on total service utilization.

We present COVID-19 effect results in terms of percent facilities relative to the predicted values to assess the magnitude of the effect relative to the situation had COVID-19 not occurred, using the following equation:

6 $$COVID-19\ effect\ perc=\left(\frac{{Use}_{t, observed}-{\widehat{Use}}_{t}}{{\widehat{Use}}_{t}}\right)\times 100$$

## Results

### National patterns of service reporting before and during COVID

**Fig. 2:**
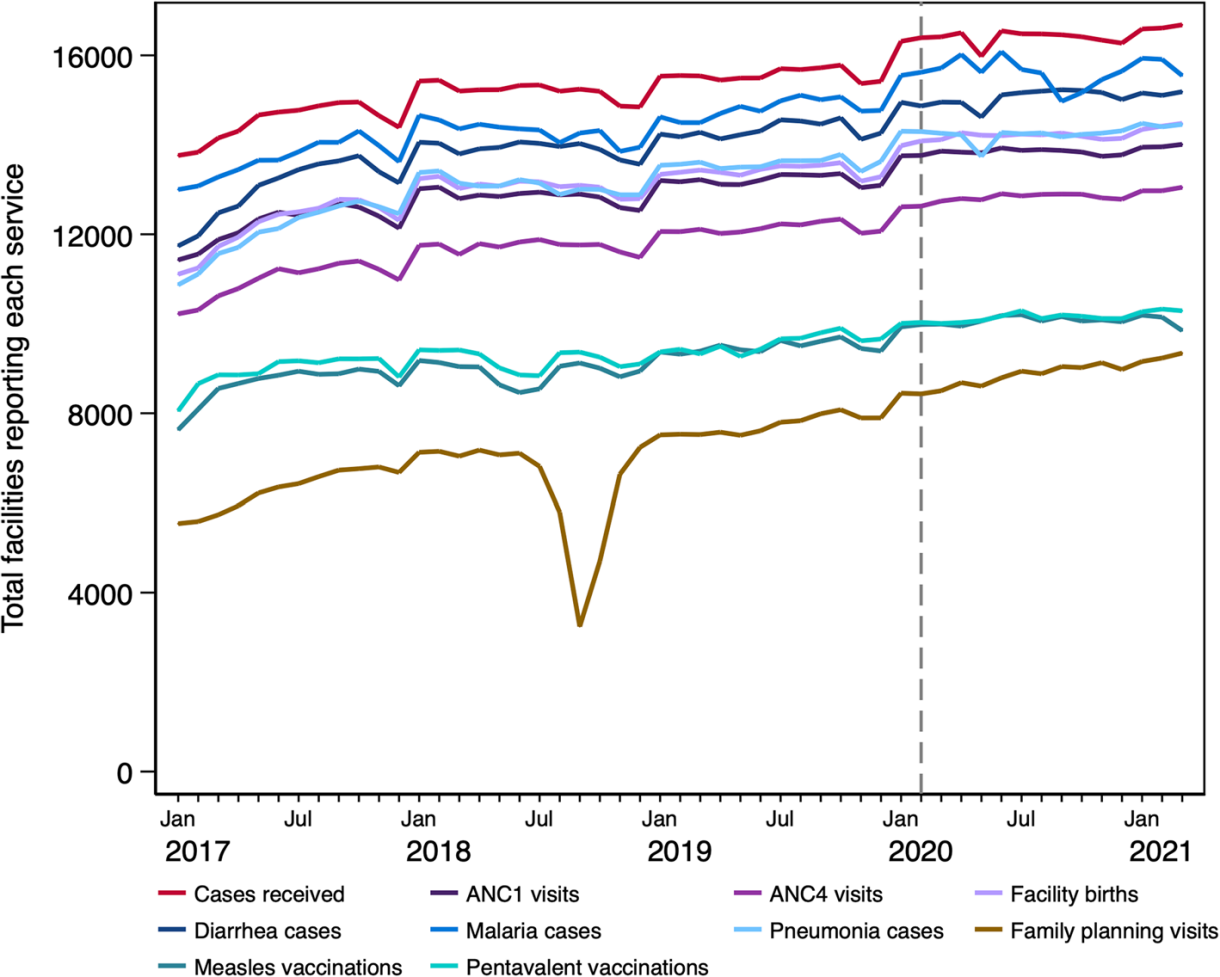
Total number of facilities reporting services during 2017–2021 in the DRC, nationally

Our initial analysis focused on the first research question: did COVID-19 affect the reporting of services to the DHIS2 database? We first examined basic patterns in the number of facilities reporting each service over time at the national level, shown in Fig. 2. There appeared to be no changes in the number of facilities reporting ANC1, facility deliveries, family planning, and immunization services during COVID-19 months. There were only small drops during May 2020 in the total number of facilities reporting any service (all cases received), diarrhea visits, malaria visits, and pneumonia visits, which mostly quickly recovered.

However, Fig. 2 only shows observed levels of reporting, and does not account for changes in health system growth during the pandemic. Therefore, in the coming sections, we also present results from our models estimating trajectories in reporting over time to further understand how COVID-19 may have affected reporting to the DHIS.

### Effects of COVID on all cases received

**Fig. 3:**
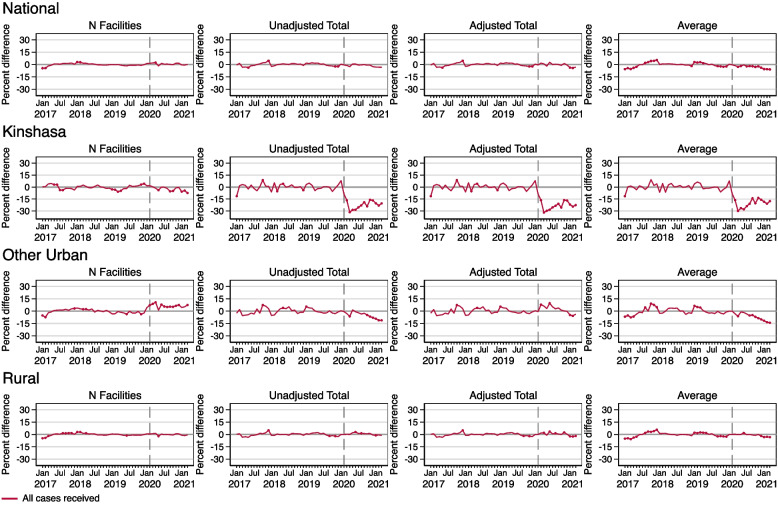
Percent difference gap between reported and predicted levels for all cases received during 2017–2021 in the DRC, nationally and by geographic area. Note: Solid circles in the graphs indicate months where the reported value is significantly different from the predicted value at *p<*0.05

Figure 3 shows the estimated COVID effects on all cases received in the DRC nationally as well as by geographic area. The first column on the left presents results from models estimating COVID-19 effects on facility reporting. There were largely no COVID-19 effects on the number of facilities reporting cases received nationally and in rural areas. In Kinshasa, there was a significant drop in the number of facilities reporting cases received in May 2020, as well as several months in late 2020 and early 2021. In other urban areas, the number of facilities reporting cases received exceeded predictions for most of the pandemic period.

In Fig. 3, the second, third, and fourth columns present results from models used to estimate COVID-19 effects on the levels of service utilization, corresponding respectively to the unadjusted total service utilization, the total service utilization adjusted for estimated levels of facility reporting during the COVID period, and average number of visits per facility. In terms of the overall service utilization levels, nationally, there were no immediate COVID effects on all cases received for either the unadjusted or the adjusted total models. Total models, adjusted for the number of facilities reporting, indicated that there may have been small and significant COVID effects in January and February of 2021 at the national level on all cases received. The average model showed small but significantly lower levels of all cases received from April 2020 through most of the entire pandemic period.

In Kinshasa, the effects of COVID on all cases received from total and average models was much larger compared to national models. For the entire first full year of the pandemic, reported levels of average and total cases received were between about 15 and 30 percent lower than predicted levels. For all three models, levels had yet to recover by March 2021. In other urban areas, unadjusted total and average models indicate that there were negative COVID-19 effects in April 2020, and then consistently after October 2020. Cases received in rural areas were largely unaffected by COVID-19, except possibly very slightly around January 2021.

### Effects of COVID on maternal and infant health services

**Fig. 4:**
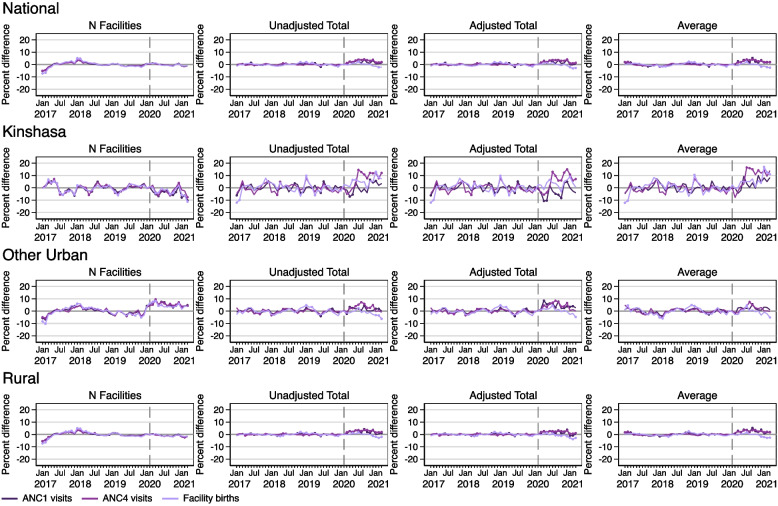
Percent difference gap between reported and predicted levels for maternal and infant health services during 2017–2021 in the DRC, nationally and by geographic area. Note: Solid circles in the graphs indicate months where the reported value is significantly different from the predicted value at *p<*0.05

Figure 4 shows results for models estimating the number of facilities reporting maternal and infant health services, as well as unadjusted total, adjusted total, and average per facility service utilization for ANC1, ANC4, and facility deliveries. There were no COVID effects on the reporting of any of the three services at the national level or in rural areas. In Kinshasa, there were negative COVID effects on reporting of these three services for most of the pandemic period. In other urban areas, the reported levels of reporting for all three maternal and infant services exceeded predictions for most of the pandemic period.

Figure 4 also shows results from models predicting levels of maternal and infant health service utilization. Nationally, COVID-19 affected antenatal care. There were periodically and consistently higher reported levels of ANC1 and ANC4, respectively, that exceeded predicted levels across all three models. There were few negative COVID effects on facility deliveries except in the average and adjusted total models during early 2021.

When investigating COVID-19 effects by area, there were some periodic negative effects in Kinshasa on total ANC1 visits. However, there were months during the pandemic where reported levels of ANC4 visits and facility deliveries were larger than predicted in all three models. In other urban and rural areas, observed total ANC1 and ANC 4 visits exceeded predictions from both the unadjusted and adjusted models for much of the pandemic period. Reported levels of facility births were generally as expected in other urban areas, except possibly in early 2021, where there are some small negative COVID-19 effects across all three models. In rural areas, there were also significant negative COVID-19 effects during late 2020 and early 2021 on facility deliveries.

### Effects of COVID on young child illness visits

**Fig. 5:**
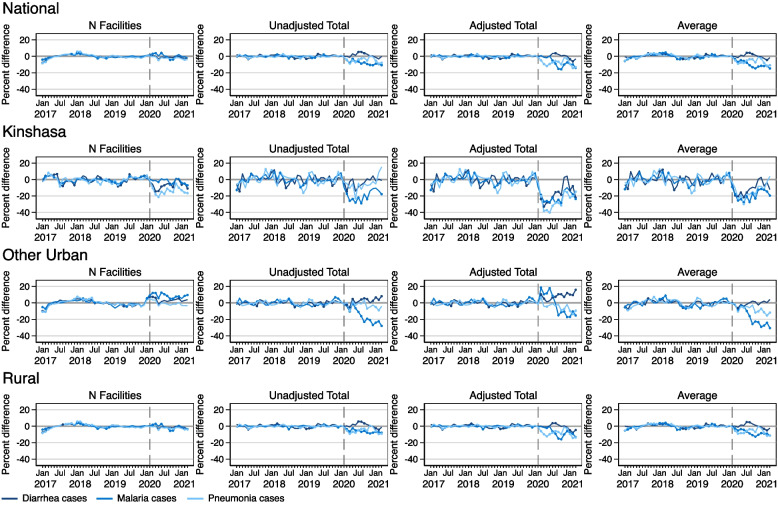
Percent difference gap between reported and predicted levels for young child visits during 2017–2021 in the DRC, nationally and by geographic area. Note: Solid circles in the graphs indicate months where the reported value is significantly different from the predicted value at *p<*0.05

Figure 5 shows results for models estimating the number of facilities reporting young child illness visits, as well as unadjusted total, adjusted total, and per facility average number of child visits for diarrhea, malaria, and pneumonia. At the national level, there were very few months with negative COVID-19 effects on facility reporting of any visits related to any of the three illness types. In Kinshasa, there were significant negative COVID-19 effects on the reporting of diarrhea and pneumonia throughout the pandemic period. There were also some significant COVID-19 effects on malaria reporting during several months starting in September 2020. The number of facilities reporting malaria in other urban areas exceeded predicted levels for most of the pandemic period, and temporarily exceeded predicted levels of diarrhea at the beginning of the pandemic period, in March and April 2020. In Rural areas, there were some significant differences between the predicted and reported levels for facilities reporting visits for all three visits, but these differences were small in magnitude.

At the national level, models indicate that COVID-19 negatively affected the total number of child visits for pneumonia and malaria during the pandemic period, as well as the average number of visits per facility. Reported levels of service utilization for malaria and pneumonia fell below predicted levels for all three models for the majority of the pandemic period and had not recovered to expected levels by March 2021. National levels of diarrhea visits among children were generally as expected for most of the pandemic period across all three models.

Examining the COVID-19 effects on young child illness service utilization by area indicated that there were varied effects of COVID-19 on diarrhea-related visits among children. In Kinshasa, adjusted total and average models indicate that there were some negative COVID-19 effects on diarrhea-related, while in other urban areas, reported levels of diarrhea reporting were largely greater than what was predicted by both total models. In rural areas, diarrhea-related service utilization was mostly unaffected by COVID-19.

There were severe COVID-19 effects on malaria care in Kinshasa, other urban, and rural areas across all three models. The most severe negative effects were in Kinshasa. There were significantly higher than predicted levels of malaria visits in other urban areas for the first few months of the pandemic, according to the adjusted total model, but then it drops significantly in subsequent months.

Similarly, observed pneumonia levels across all three models and all three geographic areas were consistently lower than predicted. Other than in April 2020, the pneumonia levels in other urban areas for the first several months of the pandemic were not significantly different than what was predicted. However, all three pneumonia models for other Urban areas showed delayed effects, with cases falling below predictions starting in October 2020. There were also significant COVID effects on pneumonia in rural areas that was similar in magnitude to what was seen in other urban areas.

### Effects of COVID on family planning

**Fig. 6:**
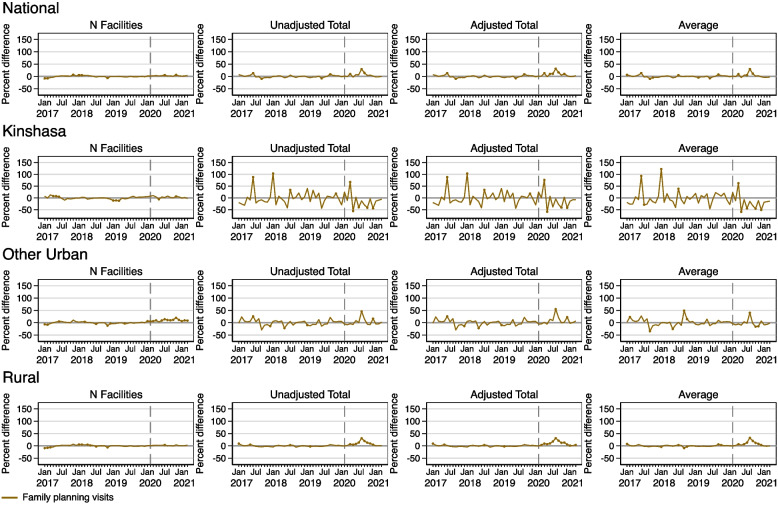
Percent difference gap between reported and predicted levels for family planning visits during 2017–2021 in the DRC, nationally and by geographic area. Note: Solid circles in the graphs indicate months where the reported value is significantly different from the predicted value at *p<*0.05

Figure 6 shows results for models estimating the number of facilities reporting family planning visits, as well as the unadjusted total, adjusted total, and per facility average. Nationally, as well as in Kinshasa and rural areas, there were no effects of COVID-19 on facilities reporting family planning. The observed number of facilities reporting family planning visits was higher than predicted for the entire pandemic period in other urban areas.

The effects of COVID-19 on the utilization of family planning services were varied. Nationally, there were some months were the reported levels of family planning utilization exceeded predicted levels in all three models, particularly in April, August, and September 2020. In Kinshasa, family planning visits met or fell below expectations during the pandemic period, except in April 2020. But these results should be interpreted with caution. There are large differences between the observed and predicted values before COVID-19, which suggest potential model instability. In other urban areas, observed family planning visits only exceeded expected levels sporadically during August 2020 in all three models. In rural areas, family planning services exceeded predictions for most of 2020, peaking in August, and largely returned to expected levels in 2021.

### Effects of COVID on child immunizations

**Fig. 7:**
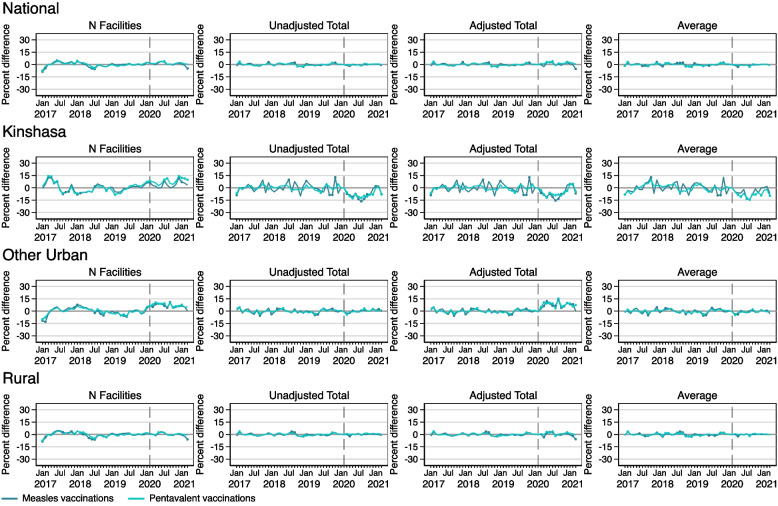
Percent difference gap between reported and predicted levels for measles and pentavalent immunizations during 2017–2021 in the DRC, nationally and by geographic area. Note: Solid circles in the graphs indicate months where the reported value is significantly different from the predicted value at *p<*0.05

Figure 7 shows results for models estimating the number of facilities reporting young child immunizations, as well as unadjusted total, adjusted total, and per facility average number of child immunizations for measles and pentavalent. Nationally and in rural areas, there were no substantial and significant COVID-19 effects on facility reporting of either type of child immunization. In Kinshasa, immunization reporting periodically exceeded predicted levels for both types during the pandemic period. In other urban areas, both immunizations consistently exceeded reported levels for most of the pandemic period.

Results from models for average and total measles and pentavalent immunizations administered nationally and in rural areas indicate that reported levels did not generally differ from predicted levels, and thus there was no effect of COVID-19. In Kinshasa, both pentavalent and measles immunizations were affected by COVID-19 during most of the pandemic period, with some evidence of recovery by January 2021. In other urban areas, unadjusted total and average models show very few COVID-19 effects on both types of immunizations, but the adjusted total shows that reported levels exceeded predicted levels for most of the pandemic period.

## Discussion

This analysis examines two main research questions on the ways the health system in the DRC was affected during the first full year of COVID-19: how did the pandemic affect reporting within the health information system and how the pandemic affected health service utilization patterns, both nationally and sub-nationally. Using DHIS2 data from the three years before the pandemic, we developed models to replicate trajectories for total and average health service utilization, in addition to the number of facilities reporting. We then extended these models to estimate levels of health service utilization had the pandemic not occurred during the first year of COVID-19 (from March 2020 to March 2021).

For the first research question, we found that COVID-19 did not affect reporting for most of the basic services we examined at the national level. However, COVID-19 could have affected reporting in Kinshasa. In the capital city, reporting was lower than expected for many services including all cases received, ANC1, facility deliveries, diarrhea, and pneumonia but higher than expected for measles and pentavalent vaccination services. This general finding, that COVID-19 had impacts on facility reporting of services, is consistent with other research. During COVID-19, there were drops in facility-level reporting in four of the eight countries included in the analysis by Shapira and colleagues [8]. A recent study using data from Bangladesh and Uganda aggregated at higher levels found no COVID-19 effects on reporting [18]. However, COVID could have still affected at lower levels, such as facilities, and been masked by the higher-level aggregation if any one facility reported a single provision of each given service.

In terms of the second research question, we found that COVID-19 affected health service utilization in the DRC. The most severe impacts among many services were experienced within the first few months of the pandemic, around the same time most governments implemented shutdown policies to prevent transmission. Nationally, the services with the most severe and lasting negative COVID effects were all cases received, young child malaria visits, and young child pneumonia visits, lacking stable recovery to expected levels. These results are consistent with previous studies also finding negative impacts on overall utilization [5–9] and disease-specific service utilization [5–7]. Additionally, levels of lower utilization among these services could have coincided with reports of COVID-19 cases and deaths, particularly among healthcare workers, which could have resulted in fears about utilizing the healthcare system. Fears among healthcare workers also may have reduced the service provision and quality as well, potentially contributing to distrust in the system [14].

Interestingly, in regard to maternal and infant health services, nationally ANC1 and ANC4 were slightly higher than expected for most of the pandemic period, while facility deliveries were expected for most of the time series and fell slightly lower than expectations during later months in the time series. Our findings for ANC1 and ANC4 visits are consistent with the findings for the DRC by similar studies [8, 13]. A study from Ethiopia also found month-to-month comparisons of ANC1 and ANC4 levels before and during COVID-19 to be similar [5]. Our findings for ANCs, however, are different than studies done in Bangladesh and Rwanda, where significant decreases were observed in ANC visits nationally [11].

During the COVID-19 period, family planning and child immunization utilization levels, which are supply-based services, were more volatile than what models predicted, mainly in Kinshasa. This volatility is also particularly prominent in the family planning service graphs in the DRC nationally. However, vaccinations were not affected very much nationally, which is also consistent with the existing research [7].

We also found that, for all services, COVID-19 effects were amplified in urban areas, particularly in the capital city, relative to the national patterns. In Kinshasa, where there were more COVID-19 cases and pandemic-related closure, negative COVID-19 effects on utilization were more severe. For many services, Kinshasa did not recover in the same manner, if at all, compared with national recovery patterns. The immediate response to the outbreak was largely concentrated in Kinshasa. The more drastic COVID-19 effects on health service utilization observed in Kinshasa likely reflect this stronger response to the outbreak.

Additionally, in the DRC, service utilization patterns during the pandemic period differed in other urban areas compared with Kinshasa or rural areas. Other urban areas in the DRC were more affected by COVID-19 than rural areas but had smaller or more delayed COVID-19 effects relative to Kinshasa. Other urban areas also had high caseloads of COVID-19, particularly during the second wave. Provinces with higher numbers of cases implemented stronger responses to control the spread of COVID-19 [14]. Geographic heterogeneity was found in other studies as well. Hategeka and colleagues investigated heterogeneity within Kinshasa and found that health service utilization was most affected in neighborhoods with the strictest lockdown measures [7]. Wanyana et al. also found geographic heterogeneity in COVID-19 effects on service utilization in Rwanda, the greatest number of services affected in the Northern and Western provinces as opposed to Kigali province. However, this study only compares the months of March and April 2019 to the same months in 2020 [11].

### Strengths

This study’s strengths help further contribute to the existing research on the impact of COVID-19 on health service utilization from LMICs. Here we analyze heterogeneity within a country, the DRC, which provides more context and is important from a practical standpoint. Analyzing the different geographic areas within the DRC is a strength unique to this analysis, as most other research on COVID-19 effects on health service utilization only examines national patterns. Within-country findings could inform policy and resource allocation at a local level. More specifically, these analyses could be used as a surveillance tool within a health system to identify disruptions, informing more timely responses by investigating and restoring affected health services.

This research continues to expand on work examining similar questions. For all selected health services in the DRC, we estimated COVID-19 effects for one full year after the start of the pandemic. Most existing research does not investigate such longer-term effects of COVID-19. Additionally, we use rigorous, but simple modelling and effect estimation strategies, that consider seasonal patterns. These models could easily be replicated as they only rely on information found within the routine health information system.

### Limitations

This analysis has some limitations concerning the models estimating COVID-19 effects. As time progresses and health systems change, the time trend patterns estimated from pre-COVID-19 data become less relevant. Therefore, there is increasing potential for error in the counterfactual predictions. Coefficients and models’ functional forms could be vulnerable to changing over time. We used models with linear time trends because they fitted the pre-COVID-19 service trajectories well, however, we could conceptualize scenarios in which the change in service utilization decreases over time, and therefore could include a negative quadratic term.

We used facility-level data for our analyses that capture smaller system disruptions but could be more prone to entry errors. Data aggregated at higher administrative facilities (e.g., districts) would be less sensitive to such errors or smaller fluctuations. However, higher-level data is less flexible in terms of model specificity. It is also harder to distinguish between types of areas (urban or rural) when using higher-level data.

Additionally, this study cannot directly attribute the changes observed to specific causes, such as access or availability of health services or resources. COVID-19 and governmental responses to the pandemic likely affected all aspects of society, and it is difficult to claim that the effects we present here were a direct result of COVID-19 itself, the governmental response, or an indirect effect caused by affordability or fear. More research is needed to understand if these changes in utilization might reflect either change in demand for or supply of health service utilization [7].

## Conclusion

Our analysis indicates that both health system reporting and health service utilization for non-COVID services in the DRC were affected by the COVID-19 pandemic. We found that the effect of the COVID-19 pandemic on service utilization varied greatly by service type and geographic location. All cases received and malaria and pneumonia visits among children under 5 were the services most affected during the first year of the pandemic. Urban areas, particularly around the capital city Kinshasa, experienced more severe impacts of COVID-19 on health services. Delayed negative impacts and persistent instability were also seen in other urban areas. However, rural areas, which were less affected by COVID-19, largely drove national patterns.

With the intent of applying such analytical approaches more widely to routine health information system data, we used a general estimation strategy instead of highly sophisticated and complex statistical modeling. Still, these simple modeling and prediction strategies are rigorous, in that they consider time trends and seasonal patterns. Beyond the pandemic context, the analytical approach used in this paper could be used as a form of surveillance to identify service disruptions and inform timely responses within health systems by policy makers.

## Supplementary Information

**Additional file 1:** **Supplementary Material S1.** Determining the functional form of time. **Supplement S2.** Assessing model fit. **Supplementary Material S3.** Hypothesis testing of COVID-19 Effects.

## Data Availability

We received authorization from the Ministry of Public Health to use these data to evaluate the impact of the pandemic on health service utilization. However, the dataset is not publicly available and researchers who wish to use these data are required to also obtain authorization from the Ministry of Public Health at the DRC.

## Abbreviations

ANC1: First antenatal visit
ANC4: Fourth antenatal visit
DHIS2: District Health Information Software version 2
DRC: Democratic Republic of the Congo
FP: Family planning
LMIC: Low and middle income country
MCH: Maternal and child health
RHIS: Routine health information system
