# Supplementary material for: Area-specific covid-19 effects on health services utilization in the Democratic Republic of the Congo using routine health information system data
Source: BMC Health Serv Res. 2023 Jun 3;23:575. doi: 10.1186/s12913-023-09547-9 (PMC10239053; doi:10.1186/s12913-023-09547-9)
Supplement: Supplementary file 1 — Additional file 1: Supplementary Material S1. Determining the functional form of time. Supplement S2. Assessing model fit. Supplementary Material S3. Hypothesis testing of COVID-19 Effects. [file 12913_2023_9547_MOESM1_ESM.docx]

# **Supplementary Material S1. Determining the functional form of time.**

We began by first graphing national time series to get a sense of the overall patterns for the services. We then examined changes between adjacent time points, which we called ‘first differences’, and the change in those first differences between time point, which we called ‘second differences’. We graphed the first differences and examine the average, standard deviation, and variances of the observed values, first, and second differences.

Examining first and second differences is a way to analyze the first and second derivatives of an underlying functional form. This is very informative for determining the functional form of the relationship between use of services and time: If the actual functional form was quadratic, then it would have the following equation, where $Y$ is the service outcome of interest and $time$ is a continuous time series variable:

$Y=\alpha_{0}+\alpha_{1}time+\alpha_{2}{time}^{2}$ (1)

The first derivative of equation (1) would be $\frac{dY}{dtime}=Y'=\alpha_{1}+{2\alpha}_{2}time$. The second derivative of the equation would be $\frac{d^{2}Y}{{dtime}^{2}}=Y''={2\alpha}_{2}$.

Instead, if the actual functional form were linear, then it would have the following expression:

$Y=\alpha_{0}+\alpha_{1}time$ (2)

which has as first derivative: $Y'=\alpha_{1}$ , a constant term, and as second derivative: $Y''=0$.

Therefore, to determine which functional form to use, quadratic or linear, we can just examine the first and second derivatives:

- If the second derivative is a zero, and the first derivative is a non-null constant, then the functional form is linear.
- If the second derivative is a non-null constant, and the first derivative changes linearly over time, then the functional form is quadratic.

We examined the empirical first and second differences of the time series for each service and

country and concluded that for all services the linear functional form was linear, not quadratic.

# **Supplement S2. Assessing model fit**

To assess model fit, we considered the model’s 𝑅2, adjusted-𝑅2, and mean square error

(RMSE) for the reporting and total service utilization models. We also generated predicted values for each pre-COVID month and examined how well the predicted trajectory replicated the observed trajectory of services. We graphed the predicted and observed time series and inspected how well the predicted series tracked the level, trend, seasonality and volatility of the series over time. In addition, we examined the average difference between $\hat{y}_{t}$predicted and $y_{t}$ observed average values in the numerator, using the following measure-of-fit:

$adapted RMSE=\sqrt{\frac{\sum_{t=1}^{38} \left( \hat{y}_{t}-y_{t} \right)^{2}}{38}}$

# **Supplementary Material S3. Hypothesis testing of COVID-19 Effects**

# When estimating the effects of COVID-19 on health services, we tested the following hypotheses:

$$H_{0}: \hat{y}_{t}=y_{t}$$

$$H_{a}: \hat{y}_{t}\neq y_{t}$$

# where *t* is any single month in the time series, $y_{t}$ is the observed value for that month, and $\hat{y}_{t}$ is the model-predicted value for that month. In short, we tested whether the predicted value and observed values at each month was significantly different.

For all hypothesis testing, we estimated test statistics at $\alpha=.05$ and corresponding 95% confidence intervals (95% CI) using standard errors ($\hat{SE}_{t}$) directly predicted from the models. However, the approach we used to estimate test statistics and 95% CI varies slightly, based on the type of dependent variable being modelled.

When modelling the number of facilities reporting as well as the unadjusted and adjusted total service utilization, there was just one value per time point. Therefore, we estimated z-scores for each month, along with p-values and 95% CI using a critical value of 1.96. The equation for the 95% CI for the predicted values for each month is as follows:

$${95\% CI}_{t}=\hat{y}_{t}\pm1.96*\hat{SE}_{t}$$

The z-score equation we used is as follows:

$${zscore}_{t}=\frac{(y_{t}-\hat{y}_{t})}{\hat{SE}_{t}}$$

Notice that the z-scores were estimated with the observed value relative to the predicted—a reverse configuration to what is customary. We did this because the predicted value in our analysis is the counterfactual value. P-values from z-tests were two-tailed and calculated using this equation:

$$P_{t}=2*(1-1.96*{zscore}_{t})$$

However, the number of facilities varied by month, which we needed to take into account when testing hypotheses on the average service utilization per facility. Therefore, a t-distribution is more appropriate for estimating p-values and 95% CIs. So when examining the significance of COVID-19 effects on average service utilization we first determined month-specific critical values (${CV}_{t}$) at $\alpha=.05$ based on the number of non-missing facilities in the dataset. The equation for the 95% CI for the predicted values for average service utilization each month is as follows:

$${95\% CI}_{t}=\hat{y}_{t}\pm{CV}_{t}*\hat{SE}_{t}$$

The t-score equation we used is as follows:

$${tscore}_{t}=\frac{(y_{t}-\hat{y}_{t})}{\hat{SE}_{t}}$$

P-values from t-tests were two-tailed and were calculated using this equation:

$$P_{t}=2*(1-{CV}_{t}*{tscore}_{t})$$
